# SCARS-LOGISTIC: A novel variable selection approach for binary classification model to identify the significant determinants of sexually transmitted infections

**DOI:** 10.1371/journal.pone.0324395

**Published:** 2025-06-09

**Authors:** Maryam Sadiq, Nasser A. Alsadhan, Ramla Shah, Sidra Younas, Zahid Rasheed

**Affiliations:** 1 Department of Statistics, University of Azad Jammu and Kashmir, Muzaffarabad, Pakistan; 2 Department of Computer Science, College of Computer and Information Sciences, King Saud University, Riyadh, Saudi Arabia; 3 Department of Statistics, Xi’an Jiaotong University, Shaanxi, Xi’an, China; The First Hospital of Jilin University, CHINA

## Abstract

Variable selection methods are very popular, especially in the field of big data with large predictors. These procedures improve the accuracy and performance of the model by eliminating irrelevant and redundant variables. The main contribution of this study is to couple a logit model with a novel variable selection approach, "Stability Competitive Adaptive Re-weighted Sampling" to address binary response. The efficiency of the proposed method is compared with the traditional logistic regression model based on eight model assessment criteria over real data from sexually transmitted infections in Indian men. Due to higher stability, the proposed method outperformed having a lower Akaike information criterion, and the Bayesian information criterion, as well as higher R-squared measures. The finally selected proposed model identified essential information regarding sexually transmitted infections in India for policymakers.

## 1 Introduction

Variable selection approaches have remained highly popular in almost every scientific field in recent decades, particularly genetics and health. The primary objective of variable selection is to decide that the final model with selected variables has minimum prediction errors [1]. Several studies concentrated on choosing significant predictors with more effective statistical methods to reduce noise and redundancy and enhance model performance. Variable selection methods are advantageous as they avoid the curse of dimensionality, decrease the complexity of the model, make the interpretations easy, and obtain an optimal model with higher performance and minimum errors [2]. Variable selection methods are applied to increase the generalization potential of a classification model. Recently, more efficient boosting methods based on logit or probit approaches with error-eliminating functions are introduced possessing higher efficiency [3,4]. Large-scale datasets with numerous predictors introduce the curse of dimensionality and multicollinearity. To establish an effective algorithm with improved prediction ability, a suitable statistical technique paired with an efficient variable selection approach is essential [5,6]. The linear regression modeling technique for continuous response and the logistic regression (LR) approach for categorical outcome remained the most widely applied method for estimation and prediction [7,8]. Most popular variable elimination methods include filter, wrapper, embedded, and recursive approaches in domains like public health, genetics, and bio-informatics [9]. Specifically in the context of logistic regression for classification, forward selection, backward elimination, and stepwise extraction are the commonly used variable selection methods. Additionally, several machine learning algorithms such as K-Nearest Neighbors (KNN), Support Vector Machines (SVM), Artificial Neural Network (ANN), Decision Tree, and Augmented Random Search (ARS) are introduced as more accurate variable selection methods in the context of regression and classification [10,11].

Numerous studies present improved feature extraction techniques for diverse data types in various fields. To handle massive data, [12] introduced the Scalable Global Mutual Information system by considering dependency among variables. Based on the redundancy elimination measure, [13] proposed a distance-based redundancy-proof for a large dataset. Particularly for classification framework, [14] suggested a distributed ensemble method for highly Skewed imbalanced big data to select an informative variable subset to enhance prediction efficiency. Filter-based methods for collinear data in the context of partial least squares are proposed for regression and classification [15,16]. [17] consider a homogeneous ensemble feature ranking procedure on medium datasets. Regression coefficients, t statistics, a hybrid of relaxed lasso and ridge regression, variable importance plots (VIP) and genetic algorithm (GA) have recently introduced improved variable selection methods [18–21]. Most recently, the competitive adaptive re-weighted sampling (CARS) method integrated with logistic regression has been established with an application of perinatal mortality data. The CARS approach step by step selects important variables by considering the absolute measure of regression coefficients that feature efficiency over calibration. The variables are selected based on weighted probabilities by executing the Monte Carlo sampling procedure, the multivariate calibration method, the exponentially decreasing function technique, and adaptive reweighted sampling [22]. The drawback of this method is to only consider the value of the coefficient estimate for determining the importance of variables [23]. The stability of the regression coefficients determined by the ratio of absolute measure and its standard deviation is considered to address this deficiency [24–26].

Hence, an improved variable selection method called stability competitive adaptive re-weighted sampling (SCARS) was presented for continuous response based on the CARS approach [27]. The present study adopted the SCARS approach for the binary classification model to identify the important risk factors of sexually transmitted infections (STI) in Indian men.

The main contributions of this research are summarized as follows. First, it integrates the SCARS algorithm with a logistic (SCARS-Logistic) model for classification. Second, the SCARS-Logistic method is compared with a classical step-wise logistic regression model using eight model assessment criteria. Further, important variables of STI in Indian men are identified using the optimal modeling strategy for future research suggestions in the medical domain. The rest of the article is organized as follows. Sect 2 describes the methods. Sect 3 presents the results followed by a discussion in Sect 4.

## 2 Materials and methods

After the validation of necessary assumptions, the standard logistic regression (LR) model integrated with the stepwise variable selection method is executed. Then, the proposed paradigm is outlined and illustrated with its outcomes.

### 2.1 Logistic regression modeling technique

Logistic regression has become a useful tool as a machine learning classification modeling strategy. It enables machine learning systems to identify category data using the fundamental equation,

$$ln(\frac{Y}{1-Y})=\alpha +\beta X+\epsilon$$ (1)

where α is the intercept, β represents the vector of estimates, ϵ indicates the residuals and the expression $\left(\frac{Y}{1-Y}\right)$ denote an odds ratio.

### 2.2 Standard variable selection method

The logistic regression coupled with the stepwise variable selection approach is frequently used to add significant variables and remove non-significant variables in the model. The proposed method is preferred over standard variable selection methods due to the advantage of application of exponentially decreasing function (EDF) to remove the least important variables based on least stability measure.

### 2.3 Stability competitive adaptive re-weighted sampling integrated with logistic regression (SCARS-Logistic)

**Fig 1:**
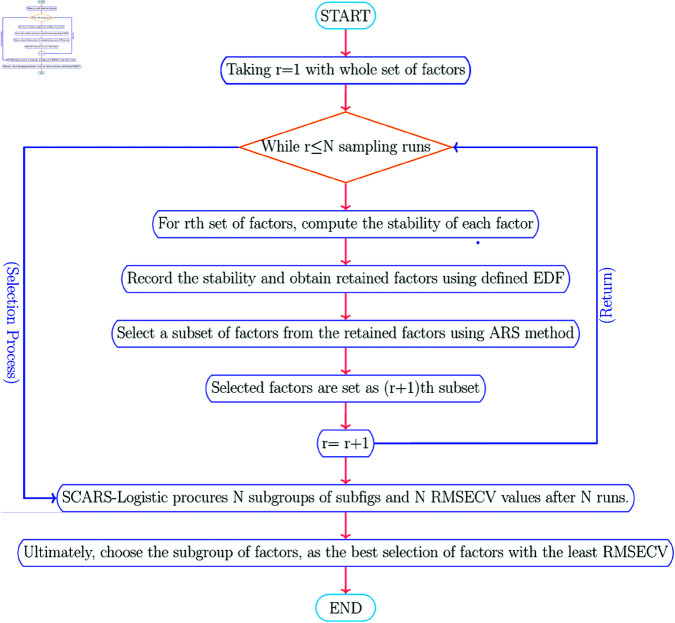
The workflow of SCARS-Logistic algorithm.

Let *X* be a n*p matrix of predictors over *n* samples and *Y* be the n*1 vector of binary response. Fig 1 shows the algorithm of the SCARS-Logistic model. The SCARS-Logistic method has many loops.

It starts with the sampling merged with the Monte-Carlo method and then stability is defined with regression coefficients (*c*_*k*_) given as:

$$s_{k}=\left|\frac{\text{mean}(c_{k})}{\text{standard deviation}(c_{k})}\right|$$ (2)

where *c*_*k*_ was the stability of ${\text{k}}^{\text{th}}$ variable in N sampling runs. The modular value ensures the positive values of *c*_*k*_. The higher value of *c*_*k*_ indicated the importance of the variable in the model.

EDF is then used to remove the variables that are comparatively less stable by force in the next stage. The ratio of variables to be kept in the ${\text{N}}^{\text{th}}$ sample run is calculated using an EDF defined as,

$$q_{r}=ze^{-tr}$$ (3)

where *z* and *t* are two constants specified by two conditions: (i) in the first sampling run, all *k* variables are used for modeling, resulting in *r*_1_ = 1,(ii) in the ${\text{N}}^{\text{th}}$ sampling run, only two variables are used for modeling, resulting in $r_{N}=\frac{2}{v}$. With these conditions *z* and *t* can be calculated as:

$$z=(\frac{v}{2}{)}^{1/N-1}$$ (4)

and

$$t=[\frac{ln(\frac{v}{2})}{N-1}].$$ (5)

Following EDF-based variable reduction, ARS is used in the SCARS-Logistic to compete to remove variables to get the best subset. This step is based on the notion of survival of the fittest. Random weighted sample experiments with replacement are used to test variables with dominant stability and retained in each scenario, whereas less competitive are discarded due to their poor stability.

The k-fold cross-validation procedure is a widely used approach to assess the performance of a machine learning algorithm or configuration on a lower-variance dataset. We used a 10-fold cross-validation to see how well the model works when different subsets of the data are selected.

Eight model assessment criteria including Akaike information criterion (AIC), Bayesian information criterion (BIC), McFadden’s Pseudo R squared ($R_{M}^{2}$), Adjusted McFadden’s Pseudo R squared ($R_{AdjM}^{2}$), Cox & Snell Pseudo R squared ($R_{CS}^{2}$), Nagelkerke/Cragg & Uhler’s Pseudo R squared ($R_{N}^{2}$), Tjur’s Pseudo R squared ($R_{T}^{2}$), and Efron’s Pseudo R squared ($R_{E}^{2}$) are applied to check the efficiency of the classical and ML techniques.

### 2.4 Data simulation for binary response

The simulated data following the binomial distribution is generated in R software having 70 predictors and a binary response with a sample size of 5000. The rbinom function generated the random values from the given sample. The probability of success ranges from 0.2 to 0.9 for variables. The simulated data are then divided into testing and training sets for further analysis.

### 2.5 Real dataset of sexually transmitted infections (STI) in Indian men

The data was acquired from the Demographic Health Survey (DHS) 2015-16 under the name ’National Family Health Survey 4 (NFHS-4)’, which was coordinated by the ’International Institute of Population Studies (IIPS) Mumbai, India, and 14 Field Agencies’ covering the population of men (aged 15 to 54 years).

A total of 61 predictors with 2817 observations having complete information are considered. The data under consideration includes 939 cases and 1878 controls showing a ratio of 1:2. The predictors are categorized into binary and multiple categories. The matrix of predictors is addressed by ***X***_***i***_ where (i = 1,2, ... ,60). The binary response ***Y*** is coded as 0 representing the absence and 1 indicating the presence of STI. Stepwise selection and SCARS coupled with logistic regression for binary classification model are executed and compared over simulated and real datasets of STI.

## 3 Results

Stepwise variable selection and SCARS coupled with logistic regression for the binary classification model are executed and compared for simulated and real datasets of STI.

### 3.1. Simulation based results

**Fig 2:**
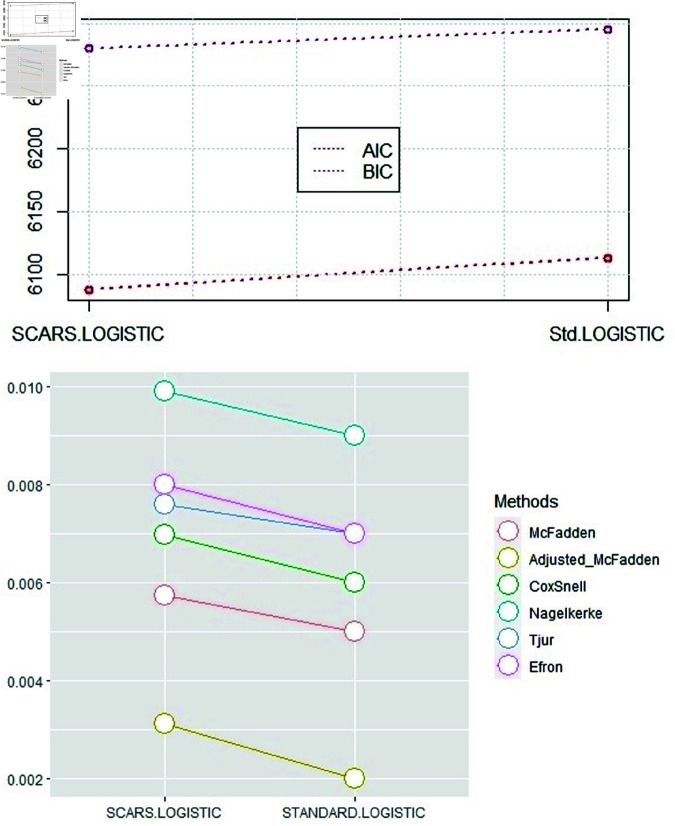
The efficiency comparison of Standard-Logistic and SCARS-Logistic variable selection methods over a simulated dataset of STI based on AIC and BIC is presented in the upper panel, the lower panel illustrates the comparison computed by ${\text{R}}^{2}$ measures.

The simulated dataset following binomial distribution is generated with 5000 observations and 70 predictors, imitating the logistic regression basic model. The dataset is then divided into testing and training sets following the 70:30 ratio to assess the efficiency of the proposed technique compared to the classical method. The charts in Fig 2 show that the SCARS-logistic model is the advantageous method of selecting variables for simulated binary data. The findings indicate that the SCARS-Logistic model surpasses the standard approach over artificially generated data from the binomial family. The SCARS-Logistic method is observed to be more efficient than the Standard-Logistic model by each model assessment criterion.

### 3.2 Real data application

**Fig 3:**
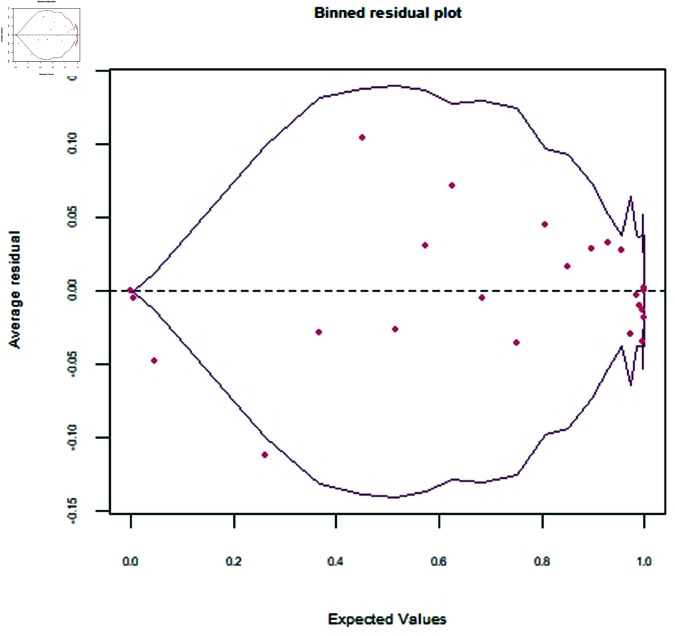
Binned residual plot.

The study obtained data on men’s health from the survey of the Indian Demographic Health Survey. The binary outcome of interest is the presence or absence of STI. Initially, 61 variables are considered and after removing zero-variance predictors, 54 are included in the analysis. The assumption about the error terms must be fulfilled to avoid misfitting the model. Fig 3 depicts the random and in-bound dispersion of error terms. The standard error bounds are set to ±0.15.

The spread and pattern of error terms indicate independent and detached behavior. Fig 3 shows the desired performance of residuals.

**Fig 4:**
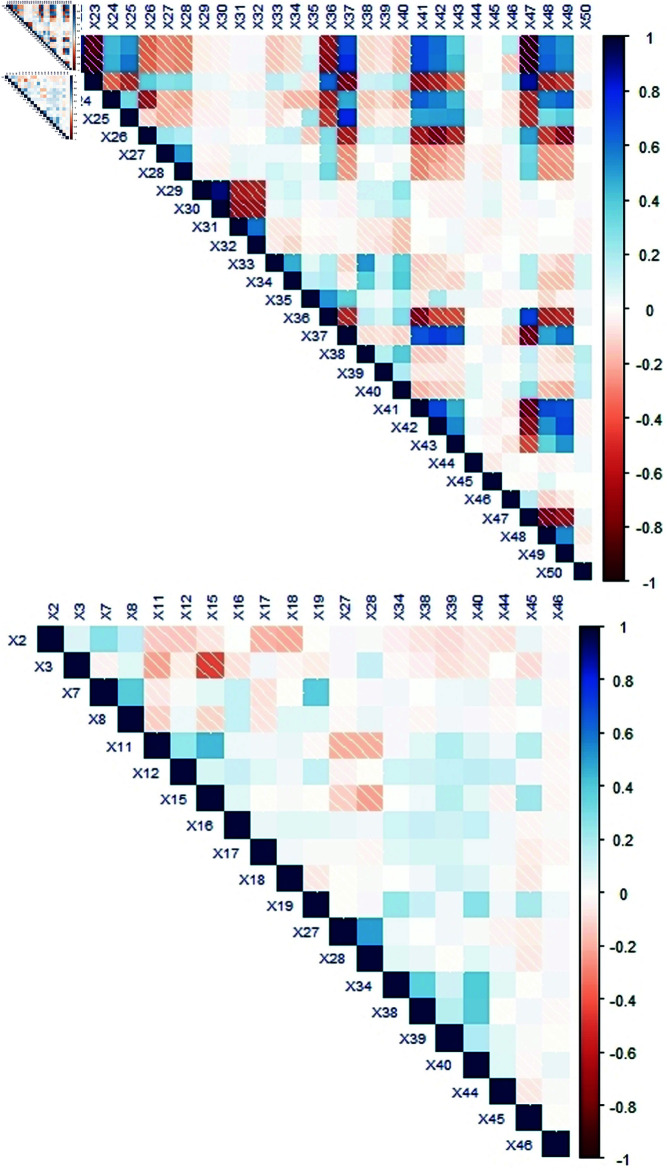
The correlation plot before removing multicollinearity is presented in the upper panel and the lower panel shows the plot after removing multicollinearity.

A correlation map displayed in Fig 4 is generated to observe the correlations between the explanatory variables. The positive and negative associations are highlighted by blue and red tints respectively in upper panel of Fig 4. Concerning multicollinearity, 15 of the 54 predictors exhibit high collinearity (>0.7). A convenient method to avoid multicollinearity is to exclude correlated explanatory variables. Then, applying the remedial measure, 39 uncorrelated explanatory variables are manifested in lower panel of Fig 4.

The final analysis is executed on 39 predictors using two selection methods (the SCARS-Logistic and the Standard-Logistic) established on eight assessment criteria.

Table 1 shows the performance of models demonstrated by the AIC, BIC, $R_{M}^{2}$, $R_{AdjM}^{2}$,$R_{CS}^{2}$, $R_{N}^{2}$, $R_{T}^{2}$, and $R_{E}^{2}$ indicating that the SCARS-Logistic is the optimal method for selection of subsets in case of binary categorical variables.

**Table 1:** Summary measures of models over STI dataset.

Methods	SCARS-Logistic	Standard-Logistic
AIC	877.15	897.0441
BIC	974.30	984.14
$R_{M}^{2}$	0.76	0.7
$R_{AdjM}^{2}$	0.75	0.7
$R_{CS}^{2}$	0.62	0.6
$R_{N}^{2}$	0.9	0.79
$R_{T}^{2}$	0.8	0.7
$R_{E}^{2}$	0.78	0.7
Number of selected variables	12	17

**Fig 5:**
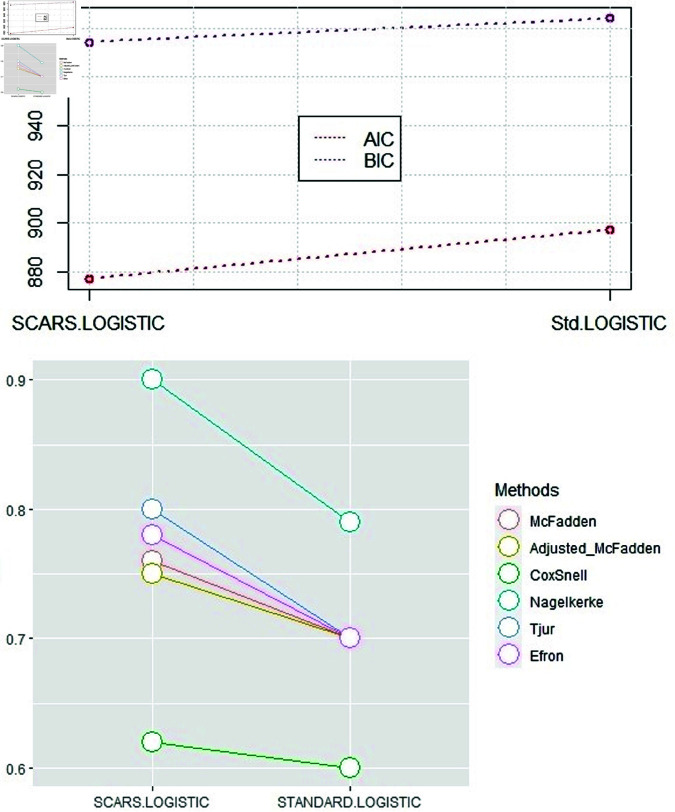
The comparison of the Standard-Logistic and the SCARS-Logistic method over a real dataset of STI based on AIC and BIC is presented in the upper panel, and the lower panel illustrates the comparison computed by ${\text{R}}^{2}$ measures.

Fig 5 shows the proficiency of models demonstrated by the AIC, BIC, $R_{M}^{2}$, $R_{AdjM}^{2}$,$R_{CS}^{2}$, $R_{N}^{2}$, $R_{T}^{2}$, and $R_{E}^{2}$. The graph in Fig 5 shows that the SCARS-Logistic model is the optimal variable selection approach for categorical variables. The optimization of the SCARS-Logistic is suggested by the highest values of Pseudo R-squared and the lowest value of AIC and BIC compared to the standard logistic method. Fig 5 shows the excellence of all methods applied in the STI data since the SCARS-Logistic performed better than the standard-Logistic approach.

**Fig 6:**
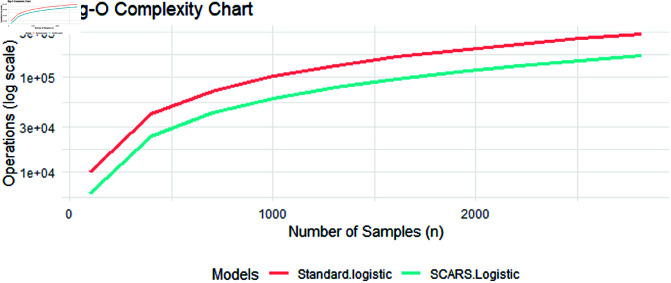
Big O comparative chart.

Fig 6 shows the Big O complexity comparison chart using the Gradient Descent method. The graph displays that the SCARS-Logistic is generally faster than standard-Logistic as its overall time complexity is smaller for each data point. In the present scenario, the SCARS-logistic performs better than standard-logistic due to low computational time.

The highly significant risk factors for STI in India and their respective regression coefficients are presented in Table 2. The findings in Table 2 indicate the notable risk factors for perinatal mortality selected by the SCARS-Logistic in common with the Standard-Logistic method.

**Table 2:** Regression coefficient estimates of the SCARS-Logistic and the Standard-Logistic method to select important variables of STI.

Risk Factors	SCARS	STANDARD
Respondent circumcised	1.1371***	1.27406***
Had genital sore/ulcer	1.7922***	1.67602***
Had genital discharge	1.9826***	2.02450***
Sex partners other than spouse	0.5847***	0.56702***
Sought help for STI infection	3.7612***	3.81002***
Knowledge about STIs	24.5404	24.93973
Multiple marriages	0.4017	0.000
Use of condom	-0.6013***	-0.48598**
Used protection in last coitus	-0.5581***	-0.42734**
Consulted doctor about STI	-0.2818	-0.49567
Awareness about HIV	0.6850*	0.92903**
Relationship to household head	-0.4531**	-0.46083**

***Highly significant, **Moderately significant, *Significant.

It is observed from Table 2 that respondents were circumcised, had genital sore/ulcer, had genital discharge, had sex partners other than the spouse, sought help for STI infection, used a condom, and used protection in the last coitus are observed to be significant variables of STIs in India regarding both variable selection methods. Multiple marriages are the risk factor selected by the SCARS-Logistic method only but dropped by the standard-Logistic method.

According to Table 2, usage of a condom, use of protection in last coitus, consulting a doctor about STIs, and relationship with the household head are negatively associated with the occurrence of STIs in the male population of India.

## 4 Discussion

The main purpose of this study is two-fold; first, to introduce an efficient variable selection method in the context of binary response coupled with logistic regression, and second to identify the significant risk factors of sexually transmitted infections in men belonging to India. Many studies are conducted to identify the causes of sexually transmitted infections (STIs) in different regions which opened up the way for numerous variable extraction methods. The current study discussed an improved variable selection method named " the SCARS-Logistic" to choose the most stable subset of variables from the larger set for higher efficiency and improved performance. For comparison of accuracy over simulated and real datasets, the Standard-Logistic approach is employed. From a set of 39 variables, the suggested technique chose 12 variables based on stability.

The SCARS approach integrated with partial least squares is proposed for continuous response compared to CARS, Monte Carlo uninformative variables elimination, and moving windows partial least squares (PLS) methods [27]. Using three different datasets of tobacco, corn, and glucose, the proposed model showed higher efficiency [28]. In a parallel manner, the SCARS method integrated with the PLS regression determined caffeine content is a significantly improved model based on the root mean square error for cross-validation [29]. Two wavelength selection techniques including CARS and SCARS coupled with PLS based on discriminant analysis are addressed in a previous research to extract the significant wavelengths. The authors observed that both methods improve the efficiency, but the SCARS-PLS approach is more efficient based on the validation performance [19]. Consistent with previous studies for various real datasets, the current study evaluated SCARS as the optimal method for a binary categorical data set of STI. Recently, [22] integrated CARS with logistic regression to identify the significant risk factors of perinatal mortality. The findings showed that logistic regression couples with the CARS algorithm perform better than the standard logistic method. In the same direction, the current study is conducted to integrate the SCARS algorithm with logistic regression.

The risk factors obtained by the present research are consistent with Indian culture and supported by various past studies. This study contributed to a new relationship of STI with the household head supported by the sociocultural background. Supported by past research, the number of sex partners including and excluding spouses, multiple marriages, respondents circumcised, and partners who used condoms are observed to be the main causes of STIs [30,31].

The current work proved that the SCARS-Logistic is the optimal method for selecting the variables with higher stability. The identified risk factors are conformable to the social settings of Indian society.

## Conclusions and future works

This study proposes the SCARS logistic regression model as a better alternative to traditional logistic methods regarding model performance and variable selection for binary data. This suggests that the SCARS logistic model possesses superior interpretational potential. Concerning the medical importance of this research, the important variables of STI identified by the SCARS logistic model can help to improve care, facilitation, and quality of health. A positive aspect of the proposed method is the evaluation of the importance of each variable through stability measures, which increases the performance of the overall model. The selection of fewer variables compared to the classical method is the drawback of this method. There are some limitations of this research. This study analyzed self-reported data that can develop different types of biases. Also, the definition of STIs declared by DHS is used in this study, neglecting the other types that may affect more comprehensive insights into the subject. In future research, the proposed method can be compared with other machine learning methods, such as CARS-logistic, ridge regression, elastic net, KNN, artificial intelligence, and neural networks, using different datasets with small and large samples.

## Public interest statement

The selection of variables is a prominent topic and an essential tool in regression modeling regarding big data. Several traditional and modified variable selection methods in the context of binary response are broadly implemented. This study used “stability competitive adaptive re-weighted sampling (SCARS)" coupled with logistic regression for addressing binary variables. This method provided a more efficient variable selection procedure for the logistic model. The practitioners may analyze logistic models integrated with the SCARS method, by using the mathematical computations provided in this article to determine the relationship between binary categorical responses and predictors.

## Supporting information

Dataset (XLSX)
